# StarMA Net: A star-shape multi-scale attention network for medical imaging classification

**DOI:** 10.1016/j.isci.2025.114214

**Published:** 2025-11-25

**Authors:** Junyang Cao, Junrui Lv, Xuegang Luo, Siyu Lai, Juan Wang, Bochuan Zheng

**Affiliations:** 1 School of Computer Science, China West Normal University, Nanchong 637000, Sichuan, China; 2 Institute of Artificial Intelligence, China West Normal University, Nanchong 637000, Sichuan, China; 3 School of Medical Imaging, North Sichuan Medical College, Nanchong 637000, Sichuan, China; 4 School of Mathematics and Computer Science, Panzhihua University, Panzhihua 617000, Sichuan, China

**Keywords:** Medical imaging, Bioinformatics, Computer modeling

## Abstract

Medical image classification is crucial for clinical diagnosis. However, medical datasets often face challenges such as limited characterization capabilities, difficulties in category differentiation, and the presence of individual differences. Although attention mechanisms can enhance feature representation, existing methods often struggle to utilize spatial information effectively and lack modeling of inter-channel interactions. We propose star-shaped multi-scale attention (StarMA). (1) It retains spatial information along different orientations within each channel through axial decomposition, enhancing the model’s perception of complex structures. (2) A star-shaped structure is employed to project feature maps into a high-dimensional nonlinear space, strengthening inter-channel interactions and improving inter-class discriminability. (3) A cross-spatial aggregation learning strategy is introduced to integrate multi-scale contextual information, improving the model’s ability to handle intra-class variability. Based on StarMA, we developed StarMA Net and conducted comparative experiments on five different datasets. Compared to advanced algorithms, StarMA Net demonstrates better effectiveness and robustness.

## Highlights

• The attention module can generate more distinguishable feature representations
• Integrating multi-scale information to enhance robustness to intra-class variations
• Enhancement of channel modeling for improved inter-class discriminative capability
• Propose a visual backbone based on the attention to medical image datasets

## Graphical abstract

**Figure:**
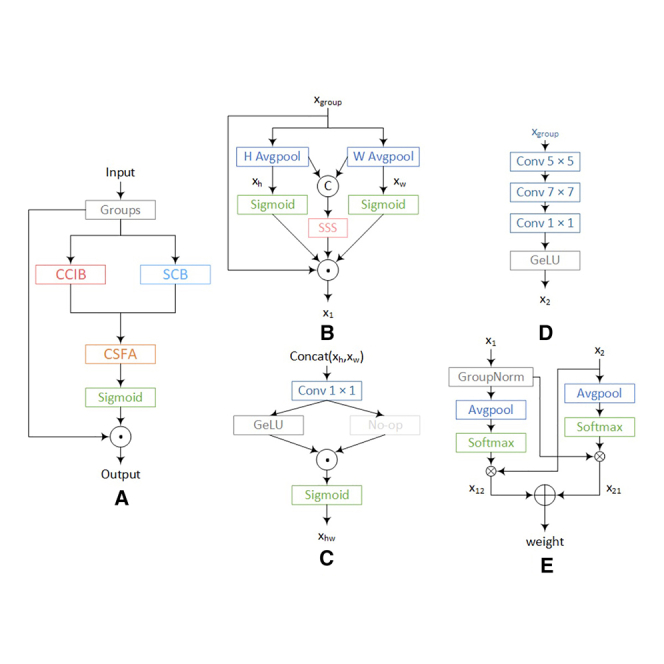

## Introduction

Medical image classification is crucial in medical imaging, significantly assisting doctors in clinical diagnosis. Mainstream diagnostic modalities such as ultrasound, endoscopy, and computed tomography (CT) play a pivotal role in diagnosing diseases early and evaluating treatment plans. Accurate classification of these medical images is essential for early disease detection and assessment of treatment efficacy. However, medical images often exhibit significant noise, and images of lesion areas frequently possess complex details, such as irregular shapes, varying sizes, and a lack of distinguishing features between categories, which contribute to the complexity of the task.^1^ Recently, deep learning has shown outstanding performance in natural image processing, prompting researchers to apply deep learning models to medical image tasks such as classification, segmentation, and target detection.^2^^,^^3^

Currently, models widely used in medical image classification fall into convolutional neural network (CNN)-based models and Transformer-based models.^4^ Both play key roles in the field of medical imaging. Computer-aided systems based on CNNs can effectively capture local information in images and have superior image classification performance. However, due to the inherent locality of the convolution kernel, CNNs focus on the fusion of local features,^5^ lacking an understanding of long-range dependencies in images, which is crucial to reflect the comprehensive information of the entire image. Attention mechanisms enable models to better capture global contextual information and effectively integrate long-range dependencies. Owing to their flexible structure, they facilitate the learning of more discriminative feature representations and can be seamlessly integrated into CNN backbones. As a result, CNN-based models incorporating attention mechanisms have been widely adopted. However, modeling cross-channel relationships through channel dimensionality reduction may have adverse effects on extracting deep visual representations.^6^

The self-attention mechanism in Transformer models effectively captures global context, encodes long-term dependencies, and has highly expressive representations.^7^ Consequently, Transformer-based models excel in image classification, segmentation, and other domains and are widely adopted. However, Transformer architectures have some limitations. Compared to CNNs, Transformer has a weaker ability to extract local information. This can lead to poor performance when the target area in an image is small, such as lesion areas in medical images. Additionally, the self-attention mechanism maps features to different spaces and constructs attention matrices through dot products, which is computationally inefficient and results in quadratic complexity with the increase in the number of tokens.^8^

Although advanced CNN-based and Transformer-based models achieve high performance on natural images, their effectiveness in medical image processing is often limited. For instance, models such as ConvNeXt^9^ and Vision Transformer^10^ have complex architectures and numerous parameters, requiring large and comprehensive datasets for training. Unlike natural images, which are abundant in quantity and variety, medical images typically suffer from limited data and significant class imbalances. Additionally, while CNN-based models can reduce complexity by employing deep separable convolutions,^11^ these decomposed convolution operations may inadequately capture the subtle and significant features present in medical images.

To address these issues, we propose a star-shaped multi-scale attention (StarMA) mechanism. Building upon StarMA, we also introduce a visual backbone network designed for the accurate classification of medical images. We conducted experiments on datasets from five different modalities, Chest X-ray, BreakHis 400X, COVID-19 CT, BUSI, and Kvasir, to assess the generalizability of our proposed method. The results indicate that our algorithm performs exceptionally well on medical image datasets across various modalities, outperforming existing CNN-based and Transformer-based backbone networks. Our contributions are summarized as follows:

1. A StarMA mechanism is proposed to capture multi-scale information, enhance feature representation, and improve model generalization, enabling accurate identification of lesions with varying shapes and sizes.
2. We consider the star-shaped structure, which enhances inter-channel information interaction and improves inter-class discriminability by mapping feature representations into a high-dimensional nonlinear space.
3. Multi-scale cross-spatial feature aggregation is implemented to generate more discriminative feature representations through cross-dimensional interactions, effectively integrating multi-scale contextual information and enhancing the model’s ability to adapt to intra-class variations.
4. Building upon the StarMA attention mechanism, we propose a visual backbone: StarMA Net, which demonstrates superior classification performance and generalization capability across five different modalities of medical imaging datasets when compared to other state-of-the-art deep learning models.

### Related work

Medical image classification is a crucial area of research for supporting clinical decision-making. This paper reviews recent advancements in the field, including the application of CNNs and Transformers to medical imaging, as well as the role of attention mechanisms and their applications in medical image analysis.

#### Applications of CNN in medical image analysis

Given the excellent performance of CNNs in natural image analysis, they are increasingly being applied to clinical-assisted diagnosis. Chen et al. established CNNs with UNet and residual neural network (ResNet) for image segmentation, from venous duplex ultrasonographic video images.^12^ Wang et al. evaluated the diagnostic performance of a multimodal deep learning (DL) model for ovarian mass differential diagnosis.^13^ Cheng et al. proposed a modular grouping attention block to capture key medical images from space, channel dimension, and their respective features, thereby improving classification performance.^14^ Cao et al. proposed an AI underdiagnosis bias discrimination pipeline for COVID-19.^15^ Jin et al. applied deep learning to achieve an accurate prediction of whole slide image (WSI) images for glioma.^16^ Wei et al. developed a 3D deep learning model to predict nonsteroidal anti-inflammatory drug efficacy for migraine.^17^ Kora et al. showed that transfer learning can achieve good results in medical image classification by fine-tuning the output of the last fully connected layer in the pre-trained ResNet-152 model.^18^ Shen et al. introduced a multi-scale crop pool strategy for DCNN to capture pulmonary nodule classification features in chest CT images.^19^ However, the convolution kernel has been focusing on local feature extraction, failing to collect enough contextual information and lacking an understanding of remote dependencies in the image, which is crucial in reflecting comprehensive details on the entire image.

#### Applications of Transformer in medical image analysis

Due to its strong capacity to capture long-range dependencies and learn global information, the Transformer is increasingly being utilized in medical image classification.^20^

Gheflati et al. used ViT to classify breast ultrasound images, and the effect was better than CNN.^21^ Sha et al. developed a hybrid CNN-Transformer-GRU model to enhance cervical lesion multi-classification.^22^ Li et al. proposed a local and global vision converter (LG-ViT) for the reconstruction of MRF parameters.^23^ Ding et al. proposed an automatic skin lesion segmentation network, named CTH-Net.^24^ Li et al. propose ViT-WSI, which is suitable for weakly supervised learning on histopathological images.^25^ Zhou et al., utilizing Transformer-based RNA sequential learning and high-order proximity preserved embedding, predict circRNA- microRNA interactions.^26^ Xu et al. designed a dual-branch encoder that combines a CNN and a Transformer to achieve accurate segmentation of skin lesions.^27^

While Transformers have made great strides in computer vision, Transformers’ self-attention mechanism often ignores local feature details. As a result, it is challenging for the model to distinguish the lesion area from the background when the lesion area in the medical image is small or the inter-class feature differentiation is low. In addition, Transformer-based models typically have numerous parameters and require extensive datasets for effective training, which is difficult to achieve for some medical images with relatively small amounts of data.

#### Attention mechanism and its applications in medical image analysis

In recent years, attention mechanisms have emerged rapidly in computer vision. Woo et al. proposed the Convolutional Block Attention module (CBAM), which is a simple and effective feedforward CNN attention module.^28^ Wang et al. proposed an efficient channel attention (ECA) module. Through appropriate cross-channel interaction, the model complexity can be significantly reduced, while the performance can be maintained.^29^ To capture pixel-level pairings and channel dependence, Zhang proposed an efficient Shuffle Attention (SA) module to solve the problem of computational overhead.^30^ Through appropriate cross-channel interaction, the model complexity can be significantly reduced while maintaining performance. Liu et al. propose a new normalization-based attention module (NAM) that suppresses less-significant weights.^31^ Hou et al. proposed coordinate attention (CA) to enhance feature aggregation by embedding spatial location information into the channel attention graph.^32^ The EMA parallel substructure proposed by Ouyang et al.^6^ helps the network avoid more sequential processing and large depth, effectively describes the learning channel, and generates better pixel-level attention to advanced feature mapping. Hu et al. proposed a new architectural unit SE, which adaptively recalibrates the channel feature response.^33^ Guo et al. propose Large Kernel Attention to avoid the limitations associated with neglecting adaptability in the channel dimension.^34^ Liu et al. utilized the dual-branch channel-spatial feature enhancement network and the coordinated attention mechanism to address the diagnostic challenges brought about by small pulmonary nodules and poor contours.^35^ Xiang et al. overcame the difficulty of processing billion-pixel images like WSI by using two-branch deep neural networks and multi-scale representation attention mechanisms to extract features directly from all patches of each WSI.^36^ Tang et al. designed a two-stream attention neural network centered on texture and shape to judge benign and malignant thyroid nodules.^37^ Fan et al. proposed an attention-based auxiliary framework for jointly localizing and classifying breast masses in ultrasound images.^38^

In recent years, attention mechanisms have achieved remarkable success in image classification. They can generate more discriminative feature representations and enhance model performance. However, while CBAM, ECA, and CA are, respectively, limited by problems such as single-dimensional interaction, spatial information deficiency, or scale imbalance, StarMA has achieved spatial-channel joint modeling, high-dimensional nonlinear mapping, and multi-scale fusion through a star-shaped multi-scale framework, significantly enhancing the ability to capture subtle lesion differences and complex structures in medical images.

To address the above issues, we propose a multi-scale attention module. This mechanism extracts subtle inter-class differences in medical images through cross-spatial feature aggregation, while leveraging a star-shaped structure to capture subtle intra-class similarities. StarMA demonstrates superior classification performance on medical imaging tasks compared to other attention mechanisms. Building upon StarMA, we developed a type of visual backbone and conducted experiments on datasets of various imaging methods.

## Results

### Materials and methods

In this section, we first introduce the StarMA module, followed by the medical image classification network StarMA Net, which is built upon it.

#### Star-shaped multi-scale attention

StarMA retains spatial information along different directions within each channel through axial decomposition, enabling the capture of shared key features across individuals and enhancing the model’s recognition ability for various shapes and sizes of lesion areas. This dynamically strengthens the model’s response to key areas and reduces the influence of irrelevant or noisy places. In the star structure of the StarMA module, we perform element-wise multiplication between the input features and the multi-scale context features, achieving explicit fusion and enhancement of the features. This operation enhances the interaction information in the channel and spatial dimensions, effectively capturing the local and global dependencies. Subsequently, we use nonlinear activation functions (such as GeLU) and learnable linear transformations to transform the node features, enabling them to obtain richer expressions in the higher-dimensional implicit feature space and fully exploit the expressive power of the high-dimensional implicit feature space.

**Figure 1:**
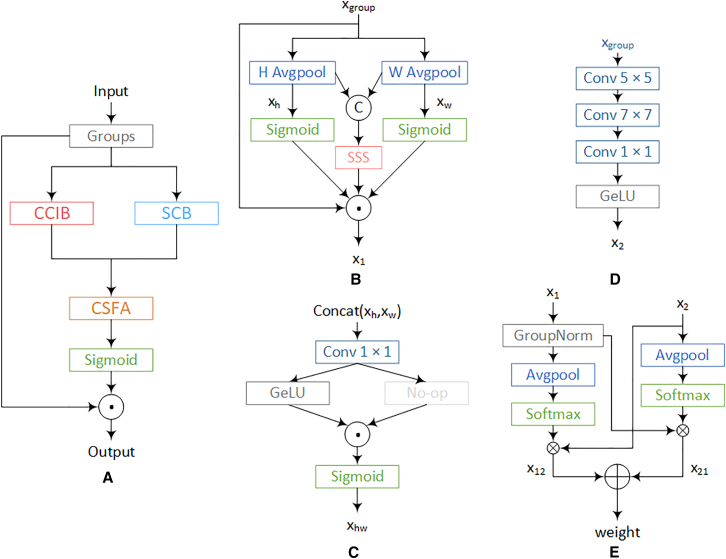
StarMA structure diagram (A) Overview of StarMA structure. (B) CCIB, cross-channel information interaction branch. (C) SSS, star-shape structure. (D) SCB, stacked convolution branch. (E) CSFA, cross-spatial feature aggregation. © Represents concat, ☉represents element-wise multiplication operation, ⊕ represents element-wise addition operation, and ⊗ represents matrix multiplication operation.

Additionally, a cross-spatial aggregation learning strategy is introduced to effectively integrate multi-scale contextual information, enhancing the model’s ability to adapt to intra-class variations while extracting more discriminative inter-class features. A schematic illustration of the proposed StarMA is shown in Figure 1. In the following, we provide a detailed description of its design.

##### Feature grouping

We first divide the input feature map X along the channel dimension into N sub-features, denoted as $x_{group}=\left[x_{0},x_{1},\ldots ,x_{N-1}\right]\;x_{i}\in R^{C//G\times H\times W}$. Each sub-feature is responsible for learning semantic information. The learned attention weight descriptors are used to reweight the feature representations of the regions of interest within each sub-feature.

##### Cross-channel information interaction branch

To capture inter-channel dependencies, we construct a cross-channel information interaction module, referred to as CCIB, to enhance the fusion and representation of multi-scale features, as shown in Figure 1B. This enables the model to effectively integrate lesion characteristics at different scales. Specifically, we perform axial decomposition to independently process two types of spatial information—along the height (H) and width (W) directions—in separate branches. This decomposition method can reduce the computational complexity, while still maintaining the modeling capability of long-term dependence. More importantly, for medical images, anatomical structures (such as blood vessels and lesion boundaries) usually extend mainly along a certain axis (vertical or horizontal). By separately modeling the H and W directions and combining them, the axial design can better capture the continuity in this direction compared to the traditional spatial attention mechanism. Lesions and tissue structures often show directional extension, and the independent modeling along the H/W direction enables the network to capture these continuous and structured patterns on a single axis. It will not be diluted by the mixed information in the other direction. It learns more refined dependency relationships in the vertical and horizontal directions, respectively, while retaining richer spatial details, thereby enhancing the model’s perception of complex structures and improving its ability to handle intra-class variations within the lesion area. Specifically, pooling kernels of size (H, 1) and (W, 1) are applied to encode the input x along the H and W directions for each channel, as shown in the Equation 1:

$$x_{h}={\text{AvgPool}}_{\left(H,1\right)}\left(x_{group}\right)$$

(Equation 1) $$x_{w}={\text{AvgPool}}_{\left(1,w\right)}\left(X_{\text{group}}\right)\text{.}$$

To establish inter-channel dependencies and enhance channel-wise interactions, a star-shaped structure is employed to improve the model’s discriminative ability in identifying lesion regions associated with different disease types. Unlike StarNet, we perform concatenation and downsizing operations on the obtained features along the h direction. Then, a star operation is adopted. At this point, the high-dimensional features contain not only height information but also width information. Each channel has complete row/column statistical features in the H + W dimension. In the subsequent 1 × 1 conv, interaction can be carried out on channels spanning h and w. The Star-shape structure is shown in the second formula in Equation 4,

$$x=concat\left(\left[x_{h},x_{w}\right]\right)$$

(Equation 2) $$x_{hw}=\text{Star}-\text{shape}\;\text{structure}\;\left(x\right)\text{.}$$

Subsequently, the Sigmoid function is applied to generate attention weights for the features extracted along the H and W directions. These weights, along with the output of the star-shaped structure, are then aggregated via element-wise multiplication to obtain cross-channel interactive features. Finally, the resulting attention map is used to reweight the input features. The detailed operation is defined in Equation 3,

(Equation 3) $$x_{1}=x_{group\;}⊙\text{Sigmoid}\left(x_{h}\right)⊙\text{Sigmoid}\left(x_{w}\right)⊙x_{hw}\text{.}$$

##### Star-shape structure

The lesion areas of some diseases often lack distinct distinguishing features, and the boundaries between classes are ambiguous. Therefore, effective recognition depends not only on spatial features but also on the subtle dependencies among the channels encoding the semantic attributes of lesions, which increases the complexity of the classification task. Therefore, we propose the SSS in Figure 1C. The high-dimensional space facilitates more effective modeling of inter-channel correlations and improves the model’s ability to capture complex feature patterns, thereby enhancing its discriminative power for lesion regions associated with different disease types. In addition, the star-shaped structure contributes to stronger modeling of inter-channel dependencies, enabling more effective information flow across channels. It further allows fine-grained weighting of different regions in the feature map, improving the model’s attention to both local and global features.

Specifically, a 1 × 1 convolution with a stride of 2 is employed for downsampling. The downsampled features are then passed through two separate branches, one of which incorporates a nonlinear activation function to increase nonlinearity. Finally, the features from both branches are fused using element-wise multiplication.

The mathematical formulation of the star-shaped structure is given by Equation4. Overall, this structure projects the features into a high-dimensional implicit feature space, enhancing the modeling of inter-channel correlations. It provides the model with richer representational capacity, enabling it to capture more complex feature patterns.

(Equation 4) $$x_{hw}=\text{Sigmoid}\left({\text{Conv}}_{1\times 1}\left(x\right)⊙\text{GeLU}\left({\text{Conv}}_{1x1}\left(x\right)\right)\right)\text{.}$$

##### Stacked convolution branch

To overcome the limitations of a single spatial scale and enable the model to capture key features shared across different individuals, thereby improving its ability to adapt to intra-class variations, a multi-scale modeling strategy is adopted in this study. Specifically, a branch, referred to as SCB, is constructed by stacking convolutional layers, as shown in Figure 1D. The SCB, in tandem with the CCIB, builds multi-scale spatial feature representations. By stacking convolutional layers with different kernel sizes (7 × 7, 5 × 5, 1 × 1), the receptive field is expanded, enhancing the model’s spatial adaptability and enabling it to extract rich contextual information. At the same time, the stacked convolutional layers progressively extract more complex and higher-level features, allowing the model to capture finer patterns and structures, thus effectively mitigating the risk of overfitting and improving generalization. Furthermore, the use of convolutional kernels of different sizes from those in the axial decomposition branch helps capture multi-scale features, allowing the model to adaptively focus on regions of interest and enhancing its robustness and adaptability in complex scenarios. Finally, the GeLU activation function is applied to introduce nonlinearity, enabling the model to learn and represent more complex spatial features, as defined in Equation 5,

(Equation 5) $$x_{2}=\text{GeLU}\left({\text{Conv}}_{7\times 7}\left({\text{Conv}}_{5\times 5}\left({\text{Conv}}_{1\times 1}\left(x_{group}\right)\right)\right)\right)\text{.}$$

##### Cross-spatial feature aggregation

To enable the model to extract and integrate spatial information across different scales, capture intra-class features, and enhance adaptability to intra-class variations, we propose the CSFA, as shown in Figure 1E. This method establishes mutual dependencies between channels and spatial locations. While preserving the local information of each spatial location, it effectively captures the global contextual information across different channels, allowing the model to more accurately capture fine-grained features and share global context between channels, thereby improving its attention to important regions.

Specifically, the output ×1 of the CCIB undergoes a Group Normalization operation, and the global spatial information is encoded using 2D global average pooling. Then, a 2D Gaussian mapping’s natural nonlinearity is applied through the Softmax function on the output of the 2D global average pooling to provide a nonlinear mapping. The resulting output is then multiplied with ×2 via matrix dot product to obtain the global spatial attention representation ×12 on the current scale.

Based on this, the output ×2 from the SCB undergoes pooling and is subjected to the Softmax function to obtain a normalized channel descriptor. The matrix dot product between this output and ×1 is then computed, generating the second attention map ×21, which retains precise spatial location information, as defined in Equation 6,

(Equation 6) $$\begin{array}{l} x_{12}=\text{Softmax}\left(\text{AvgPool}\left(\text{GroupNorm}\left(x_{1}\right)\right)\right)⊗x_{2}\\ x_{21}=\text{Softmax}\left(\text{AvgPool}\left(x_{2}\right)\right)⊗x_{1} \end{array}\text{.}$$

Finally, the two generated spatial attention weights from each group are aggregated to produce the output feature map. Subsequently, the Sigmoid function is applied to create the weights, resulting in the final attention weight descriptor, and it is multiplied by the input to obtain the final output:

$${weight=x}_{12}+x_{21}\text{,}$$

(Equation 7) $$Output=\text{Sigmoid}\left(weights\right)⊙x_{group}\text{.}$$

To more clearly illustrate the specific process of the scale fusion algorithm, we have written the pseudo-code as Algorithm 1.

Algorithm 1 **The algorithmic process of scale fusion**

Input: x1, x2

 x1 is the output of the CCIB path

 x2 is the output of the SCB path

Output: weight

 weight is the final attention map

1. x1 ← GroupNorm(x1)

2. x2 ← x2

3. x11 ← Softmax(AvgPool(GroupNorm(x1)))

4. x22 ← Softmax(AvgPool(x2))

5. x12 ← x11 ⊗ x2

6. x21 ← x22 ⊗ x1

 ⊗ represents matrix multiplication

7. weight ← x12 + x21

Output: weight

#### Network structure

**Figure 2:**
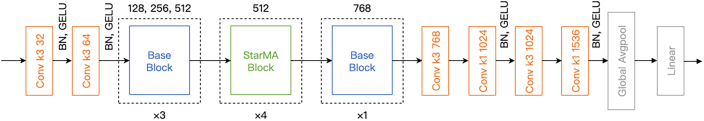
Overall structure of the StarMA Net

CNNs have achieved significant success in computer vision tasks such as image classification and object detection. However, their performance tends to degrade rapidly in complex tasks involving low-resolution images or small objects.^39^ Transformer-based models, on the other hand, typically require large amounts of data for effective training. In contrast to the abundant natural images, most medical images are limited in quantity. Utilizing such high-complexity networks often leads to overfitting on the training set, resulting in poor model robustness and generalization. Therefore, we have designed a visual backbone to enable the accurate classification of multiple medical image datasets, as shown in Figure 2.

The model consists of three components: an input stream, intermediate layers, and an output stream. The input stream includes two convolutional layers and three Base Blocks, performing four stages of spatial resolution reduction. Specifically, the input image is first processed by two convolutional layers with 3 × 3 kernels to perform downsampling, extract local features, and enhance training stability.

**Figure 3:**
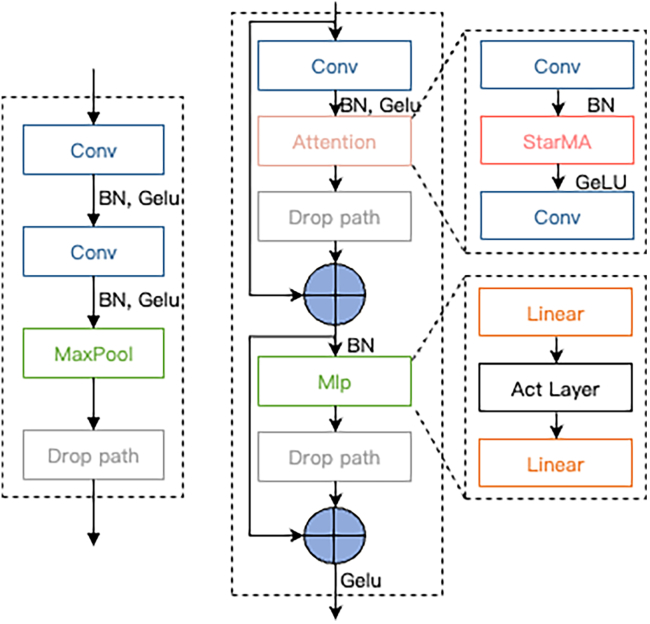
Base Block and StarMA Block ⊕ Represents adding by elements.

Subsequently, three Base Blocks are employed for further downsampling. Each Base Block is composed of a 3 × 3 convolution, followed by Batch Normalization and a GELU activation function, as illustrated in Figure 3 left. A max pooling layer is used to reduce the size of the feature maps while preserving essential features, and a Drop Path layer is embedded to prevent overfitting.

The intermediate layers of the model are composed of four StarMA Blocks, responsible for feature extraction. At this stage, no downsampling is performed, and the input and output dimensions remain consistent across all layers. Specifically, each StarMA Block first applies a 3 × 3 convolution to the input x, and the resulting feature maps are subsequently passed to the attention module for further processing. The structure of the StarMA Block is illustrated in Figure 3 (right).

The attention layer consists of two 3 × 3 convolutional layers followed by a StarMA module. After processing through the attention layer, the output is denoted as *x*_*a*_

(Equation 8) $$x_{a}={\text{Conv}}_{3\times 3}\left(\text{GeLU}\left(\text{StarMA}\left(\text{BN}\left({\text{Conv}}_{3\times 3}\left({\text{Conv}}_{3\times 3}\left(x\right)\right)\right)\right)\right)\right)\text{.}$$

By utilizing the weight calculation of multi-scale attention, the model can dynamically emphasize the feature responses of different spatial positions and channels. Based on the calculated attention weights, weighted summation or element-by-element multiplication of multi-scale features is performed to dynamically enhance the model’s response to key regions and reduce the influence of irrelevant or noisy regions.

To enhance the model’s generalization capability, we introduced a Drop Path layer in the StarMA Block. By randomly discarding certain branches, this approach helps to mitigate the problem of model overfitting. The *D*_*A*_ is *x*_*a*_ after passing through the Drop Path layer. The original input *x* of *D*_*A*_ element is added by element, to get the output:

(Equation 9) $$x_{attn}=D_{A}+x$$

In this module, we employ a multi-layer perceptron to perform nonlinear mapping and transformation on the input features to obtain *M*. This process facilitates the model in learning abstract representations of the features, thereby enhancing its ability to capture higher-level features and patterns within the input data. The output *M*, after passing through the Drop Path layer, is transformed to *D*_*M*_. Finally, *D*_*M*_ is elementwise added to*x*_*attn*_, followed by the GELU activation function to introduce nonlinearity. The resulting output constitutes the final output of the StarMA layer, as illustrated in Equation 10:

(Equation 10) $$StarMA\;Block\;Output=\text{GeLU}\left(D_{M}+x_{attn}\right)\text{.}$$

The output stream of the network begins with a Normal Block, which further refines the features produced by the StarMA Blocks to enhance feature representation and improve the model’s discriminative capability. This is followed by a sequence of a 3 × 3 convolutional layer, a 1 × 1 convolutional layer, a Batch Normalization layer, and a GELU activation function, which together perform additional downsampling. This process facilitates the extraction of more meaningful features while reducing the spatial dimensions of the feature maps, thereby lowering computational complexity and improving efficiency for the subsequent global average pooling and fully connected layers.

### Experiment and results

To evaluate the effectiveness and generalization capability of the StarMA Net model, we conducted a series of experiments on five public medical image datasets (COVID-19 CT,^40^ BreakHis 400,^41^ Chest X-ray,^42^ BUSI,^43^ and Kvasir Dataset^44^) across different modalities. In the following sections, we will present and analyze the experimental results of our method on five datasets. Additionally, to ensure the performance of our attention mechanism, we conducted a series of ablation experiments to verify the effectiveness of the StarMA we proposed.

#### Experimental results

To evaluate the performance of the StarMA Net model, we compared it with several state-of-the-art models from recent years, including VGG19,^45^ Vision Transformer,^10^ Swin Transformer,^46^ EfficientNet,^47^ ResNet,^48^ ConvNeXt,^9^ Biformer,^49^ FocalNet,^50^ UniFormer,^51^ SGFormer,^52^ FasterNet,^53^ StarNet,^8^ and HiFuse,^54^ which perform well on medical images. The training parameters were consistent across the five datasets, and the highest validation accuracy was recorded for testing. The results from all test sets were summarized and compared to assess the effectiveness of the proposed network.

##### Result on COVID-19 CT

**Figure 4:**
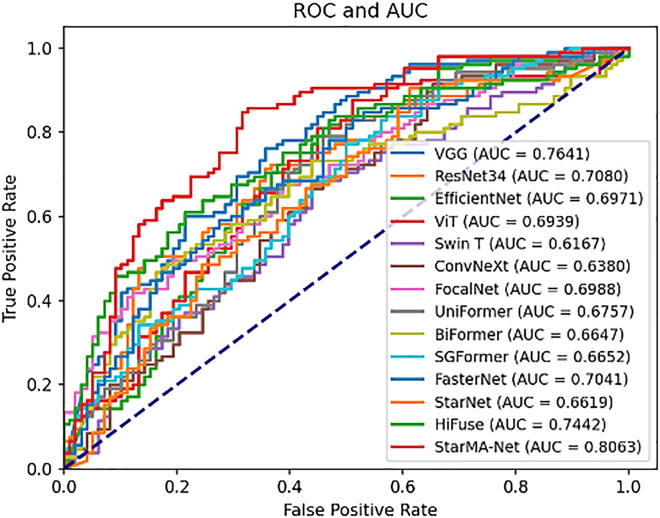
AUC-ROC curve on COVID-19 CT dataset

We applied the data partitioning method described in the literature^41^ to divide the dataset. Table 1 summarizes the evaluation of our proposed model and other advanced models on the COVID-19 CT dataset. Table 1 shows that network architectures optimized for large datasets, such as Swin Transformer and ConvNeXt, did not perform well on this dataset. Although Vision Transformer exhibited high sensitivity, its specificity was only 0.5102. In contrast, smaller models like FasterNet and StarNet demonstrated more balanced performance. HiFuse, which performs well on medical images, also demonstrates good robustness. Our proposed model achieved classification accuracy and sensitivity of 0.7241 and 0.7238, respectively. Compared to other models, StarMA Net produced more balanced predictions and was less affected by category imbalances. All evaluation metrics for StarMA Net performed optimally, indicating that it excels in medical image classification with a small amount of data and high image complexity. This demonstrates that our proposed StarMA module effectively establishes short- and long-term dependencies through multi-scale parallel subnetworks; maps inputs to high-dimensional, nonlinear feature spaces; and captures subtle differences and critical information in lesion areas, thereby enhancing model classification performance. The comparison of the area under the curve (AUC) of each model on COVID-19 CT dataset is shown in Figure 4.

**Table 1:** Summary table of results on COVID-19 CT dataset

Model	Accuracy	Precision	Sensitivity	Specificity	F1-score	AUC
VGG	0.6995	0.6864	0.7714	0.6224	0.7265	0.7641
ResNet	0.6453	0.6279	0.7714	0.5102	0.6923	0.7080
EfficientNet	0.6650	0.6276	**0.8667**	0.4490	0.7280	0.6971
Vision Transformer	0.6749	0.6444	0.8286	0.5102	0.7250	0.6939
Swin Transformer	0.5961	0.5906	0.7143	0.4694	0.6466	0.6167
ConvNeXt	0.6059	0.6000	0.7143	0.4898	0.6522	0.6380
FocalNet	0.6256	0.6559	0.5810	0.6735	0.6162	0.6988
UniFormer	0.5616	0.6111	0.4190	0.7143	0.4972	0.6757
BiFormer	0.6355	0.6505	0.6381	0.6327	0.6442	0.6647
SGFormer	0.6355	0.6084	0.8286	0.4286	0.7016	0.6652
FasterNet	0.6207	0.6186	0.6952	0.5408	0.6547	0.7041
StarNet	0.6158	0.6154	0.6857	0.5408	0.6486	0.6619
HiFuse	0.6897	0.7333	0.6286	0.7551	0.6769	0.7442
StarMA Net	**0.7241**	**0.7379**	0.7238	**0.7245**	**0.7308**	**0.8063**

The bolded items are the best indicators.

##### Result on BreakHis 400×

**Figure 5:**
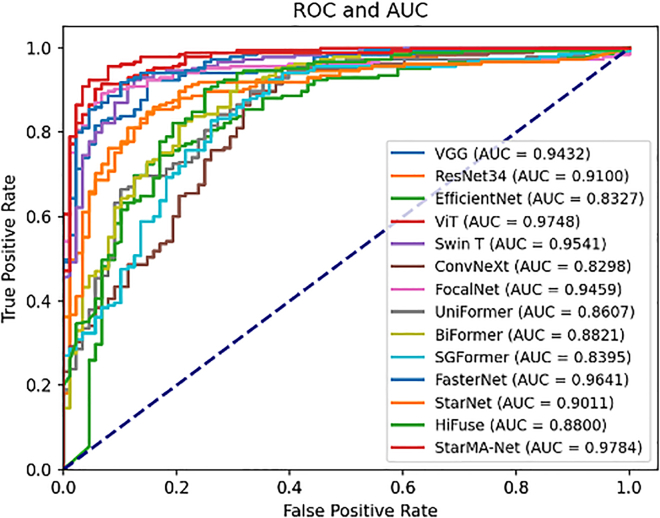
AUC-ROC curve on BreakHis 400X dataset

To further assess the generalization capabilities of StarMA Net, we conducted experiments using the BreakHis 400× dataset and summarized the results in Table 2. Unlike the COVID-19 CT dataset, which contains fewer than 800 images, BreakHis 400× includes 1,820 images. Although the dataset exhibits some class imbalance, it better aligns with the conditions required for training deep learning models. The results indicate that, with the increased data volume, all models demonstrated strong classification performance, even without resampling. Notably, StarMA Net achieved the highest F1 score and AUC on this dataset, with values of 0.9542 and 0.9784, respectively. This demonstrates that the StarMA Net has excellent generalization ability, and its classification performance also shows outstanding robustness on the pathological medical image dataset. The comparison of the AUC of each model on BreakHis 400X dataset is shown in Figure 5.

**Table 2:** Summary table of results on BreaKHis 400X dataset

Model	Accuracy	Precision	Sensitivity	Specificity	F1-score	AUC
VGG	0.8791	0.9270	0.8919	0.8523	0.9091	0.9432
ResNet	0.8315	0.8564	0.9027	0.6818	0.8789	0.9100
EfficientNet	0.7985	0.8316	0.8811	0.6250	0.8556	0.8327
Vision Transformer	0.9121	0.9215	0.9514	0.8295	0.9362	0.9748
Swin Transformer	0.9084	0.9396	0.9243	0.875	0.9319	0.9541
ConvNeXt	0.8242	0.8341	0.9243	0.6136	0.8769	0.8298
FocalNet	0.8974	0.9243	0.9243	0.8409	0.9243	0.9459
UniFormer	0.8278	0.8255	0.9459	0.5795	0.8816	0.8607
BiFormer	0.8132	0.8602	0.8649	0.7045	0.8625	0.8821
SGFormer	0.8242	0.8186	0.9514	0.5568	0.8800	0.8395
FasterNet	0.8901	0.9016	0.9405	0.7841	0.9206	0.9641
StarNet	0.8681	0.8901	0.9189	0.7614	0.9043	0.9011
HiFuse	0.8535	0.8680	0.9243	0.7045	0.8953	0.8800
StarMA Net	**0.9377**	**0.9516**	**0.9568**	**0.8977**	**0.9542**	**0.9784**

The bolded items are the best indicators.

##### Result on Chest X-ray

**Figure 6:**
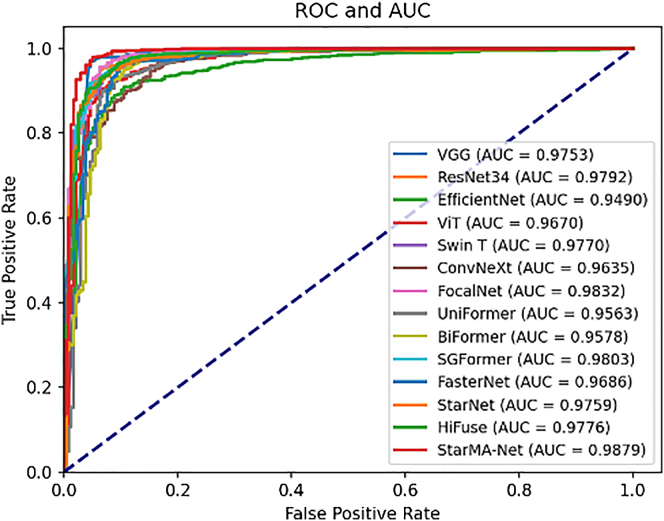
AUC-ROC curve on Chest X-ray dataset

The Chest X-ray dataset comprises 5,856 images, with more than 1,000 images per class used during the training phase. As illustrated in Table 3, the models achieve accuracies above 0.9, with some models such as VGG19 and FocalNet reaching accuracies of 0.958 and 0.957, respectively. As the data volume increases, network architectures optimized for large datasets, such as BiFormer, also perform well. Although StarNet and Faster models are smaller, they achieve performance comparable to those of models like Swin Transformer and BiFormer. HiFuse also demonstrated good robustness on this dataset, with an F1 score reaching 0.9666. Nevertheless, our proposed model outperforms all others on this dataset, achieving an accuracy of 0.9692. This demonstrates that our model performs exceptionally well even on relatively large datasets, further validating its strong generalization capabilities. The comparison of the AUC of each model on the Chest X-ray dataset is shown in Figure 6.

**Table 3:** Summary table of results on chest X-ray datasets

Model	Accuracy	Precision	Sensitivity	Specificity	F1-score	AUC
VGG	0.9579	0.9576	0.9860	0.8819	0.9716	0.9753
ResNet	0.9419	0.9456	0.9766	0.8481	0.9609	0.9792
EfficientNet	0.9009	0.9248	0.9407	0.7932	0.9327	0.9490
Vision Transformer	0.9248	0.9499	0.9470	0.8650	0.9484	0.9670
Swin Transformer	0.9442	0.9582	0.9657	0.8861	0.9619	0.9770
ConvNeXt	0.9248	0.9376	0.9610	0.8270	0.9492	0.9635
FocalNet	0.9567	0.9603	0.9813	0.8903	0.9707	0.9832
UniFormer	0.9260	0.9350	0.9657	0.8186	0.9501	0.9563
BiFormer	0.9374	0.9413	0.9750	0.8354	0.9579	0.9578
SGFormer	0.9476	0.9501	0.9797	0.8608	0.9647	0.9803
FasterNet	0.9385	0.9454	0.9719	0.8481	0.9585	0.9686
StarNet	0.9419	0.9443	0.9782	0.8439	0.9609	0.9759
HiFuse	0.9510	0.9614	0.9719	0.8945	0.9666	0.9776
StarMA	**0.9692**	**0.9709**	**0.9875**	**0.9198**	**0.9791**	**0.9879**

The bolded items are the best indicators.

##### Results on BUSI

BUSI is a three-classification ultrasound image with a small overall sample size and a lack of representative characteristics between benign and malignant nodules, and the image complexity is high, making classification difficult. Table 4 presents the evaluation of our proposed model alongside other advanced models on the BUSI dataset. ConvNeXt shows limited classification performance on this dataset. Similarly, HiFuse, SGFormer, and others based on Transformer also performed poorly on this dataset. In contrast, the smaller and more compact StarNet performs relatively well, while ResNet34 and VGG19 achieve better results. StarMA Net, however, demonstrates superior performance with a classification accuracy of 0.8120 and an F1 score of 0.7892 on BUSI dataset. Although StarMA Net has achieved the best results on the BUSI dataset, there is still room for further optimization. When examining failed cases, we observed that misclassification was often associated with nodules exhibiting blurred or indistinct boundaries, as well as the presence of ultrasound-specific artifacts (such as acoustic shadows and speckle noise), which obscured key diagnostic features. In addition, some nodules exhibit complex internal textures similar to those of the surrounding tissues, which poses a challenge to discriminative feature extraction.

**Table 4:** Comparison of performance on BUSI dataset

Model	Accuracy	Precision	Recall	F1-score
VGG	0.7265	0.7139	0.6652	0.6828
ResNet	0.7179	0.6847	0.6367	0.6456
EfficientNet	0.6410	0.5390	0.5106	0.5132
Vision Transformer	0.6667	0.6167	0.5606	0.5767
Swin Transformer	0.6581	0.6099	0.5726	0.5780
ConvNeXt	0.5812	0.4717	0.4522	0.4452
FocalNet	0.7436	0.7191	0.6348	0.6604
UniFormer	0.641	0.5736	0.5513	0.5595
BiFormer	0.7009	0.6493	0.6558	0.6522
SGFormer	0.6923	0.735	0.5468	0.5686
FasterNet	0.6239	0.5742	0.5182	0.5337
StarNet	0.6752	0.6354	0.6294	0.6279
HiFuse	0.6667	0.6309	0.5724	0.5916
StarMA Net	**0.8120**	**0.8345**	**0.7620**	**0.7892**

The bolded items are the best indicators.

Therefore, in our future work, we will focus on enhancing multi-scale feature fusion and combining edge-preserving modules to better capture the morphology of nodules. In addition, we will also attempt to integrate ultrasound-specific denoising technology and adversarial training strategies to enhance the robustness against imaging artifacts. And the utilization of context semantic information and domain adaptation methods can help alleviate the differences caused by different acquisition devices or imaging protocols, thereby improving the classification performance in different clinical environments.

**Figure 7:**
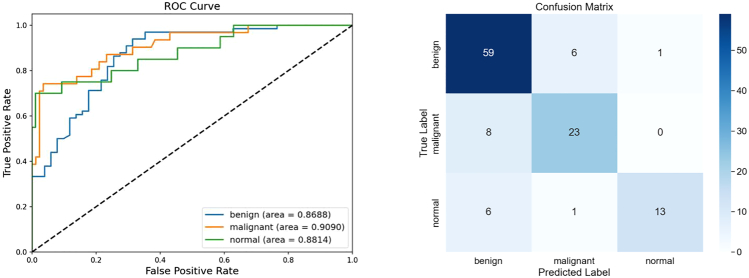
AUC-ROC curve and confusion matrix of StarMA Net on the BUSI dataset

Furthermore, since this experiment only performed division (training set, validation set, test set) and normalization operations on the dataset, and there were 133 normal samples, 437 benign samples, and 210 malignant samples in the dataset, with significant differences in the number of categories, if preprocessing operations such as reuse and data augmentation are adopted in clinical practice, better results can be achieved. We also plotted the receiver operating characteristic (ROC) curve and confusion matrix of StarMA Net. The AUC-ROC curve and confusion matrix of StarMA Net on the BUSI dataset are shown in Figure 7. As can be seen from the ROC curve, the model demonstrates excellent discriminatory ability for all categories, with AUC values exceeding 0.86 in all cases. Among them, the AUC of malignant category was the highest (0.9090) and its classification performance was the best. The AUCs of the normal and benign categories were 0.8814 and 0.8688, respectively, indicating that the model has good diagnostic value for all three types of lesions. The confusion matrix also shows that the overall classification effect of the model is good, with 59 correct benign categories. The accuracy of the malignant category is the highest, and it has strong reliability. The main misclassification of the model is concentrated in the mutual judgment between benign and malignant categories. Subsequently, the discrimination between the two categories can be further strengthened.

##### Result on Kvasir

**Figure 8:**
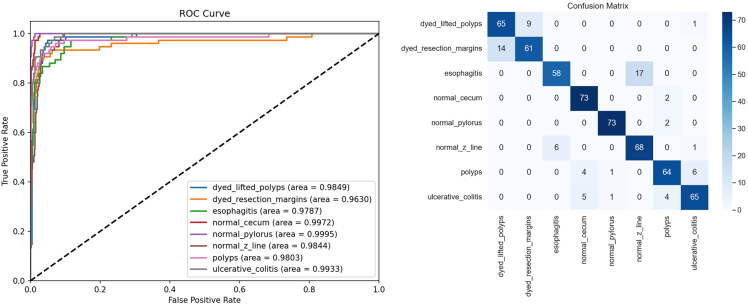
AUC-ROC curve and confusion matrix of StarMA Net on the Kvasir dataset

All the datasets exhibit class imbalance issues, so we conducted additional tests using the Kvasir dataset. The Kvasir dataset consists of 8 categories, each with an equal number of 500 images. This test aimed to assess whether StarMA Net could maintain its high performance on a class-balanced dataset. The results, presented in Table 5, indicate that, in a scenario with balanced categories, all models perform well. StarMA Net achieved classification accuracy and F1 scores of 0.8783 and 0.8778, respectively, outperforming other advanced models. These findings demonstrate that our model possesses strong robustness and generalization capabilities. We also plotted the ROC curve and confusion matrix of this dataset to evaluate the classification performance of the model. In the ROC graph, the AUC of normal_pylorus reaches 1.000 and the classification is nearly perfect. normal_cecum AUC is 0.997, and the recognition accuracy is also very high. The AUCs of other categories such as dyed_lifted_polyps (0.985) and esophagitis (0.979) are also at a relatively high level. Overall, the model performed exceptionally well in these medical image category classification tasks. It can be seen from the confusion matrix that the main diagonal values of categories such as normal_cecum and normal_pylorus account for a high proportion and the classification accuracy is high. However, there are also some misjudgments, mainly concentrated between the dyed_lifted_polyps and dyed_resection_argins categories and the esophagitis and normal_z_line categories. Subsequently, the model can be optimized for these easily confused categories to improve the discrimination. The AUC-ROC curve and confusion matrix of StarMA Net on the Kvasir dataset are shown in Figure 8.

**Table 5:** Comparison of performance on Kvasir dataset

Model	Accuracy	Precision	Recall	F1-score
VGG19	0.7783	0.7767	0.7783	0.7763
ResNet34	0.7767	0.7756	0.7767	0.7755
EfficientNet b3	0.7183	0.7282	0.7183	0.7188
Vision Transformer	0.7933	0.8013	0.7933	0.7898
Swin Transformer	0.7783	0.7829	0.7783	0.7756
ConvNeXt	0.7300	0.7272	0.7300	0.7253
FocalNet	0.8000	0.8046	0.8000	0.7972
UniFormer	0.6917	0.6990	0.6917	0.6891
BiFormer	0.7433	0.7446	0.7433	0.7396
SGFormer	0.7533	0.7737	0.7533	0.7411
FasterNet	0.7633	0.7666	0.7633	0.7609
StarNet	0.7200	0.7184	0.7200	0.7115
HiFuse	0.7983	0.8057	0.7983	0.7948
StarMA Net	**0.8783**	**0.8804**	**0.8778**	**0.8778**

The bolded items are the best indicators.

##### Comprehensive analysis of experimental results in multi-class classification tasks

We further compared ROC curves, confusion matrices, Matthews correlation coefficient (MCC), Kappa, and AUC of the proposed model against other advanced classification models on the BUSI and Kvasir datasets for a more comprehensive evaluation. As shown in Table 6, StarMA Net outperforms other models in terms of MCC, Kappa, and AUC on both the BUSI and Kvasir datasets. We show the AUC-ROC curve and confusion matrix of each model on BUSI and Kvasir datasets in the additional material. From the confusion matrix for the BUSI dataset, it is evident that StarMA Net exhibits superior capability in distinguishing normal category images compared to other models. For the Kvasir dataset, classification errors were primarily concentrated in the categories of dyed_lifted_polyps and dyed_resection_margins. StarMA Net demonstrated improved classification performance in these categories, attributed to its ability to capture subtle differences and critical information about lesion areas. This demonstrates that our model exhibits superior robustness and generalization.

**Table 6:** Comparison of MCC, Kappa, and AUC performance for various models on the BUSI and Kvasir datasets

	BUSI			Kvasir		
	MCC	Kappa	AUC	MCC	Kappa	AUC
VGG	0.5164	0.5135	0.8756	0.7470	0.7467	0.9746
ResNet	0.5029	0.4984	0.8305	0.7450	0.7448	0.9726
Efficient Net	0.3425	0.3334	0.7895	0.6794	0.6781	0.9605
Vision Transformer	0.3943	0.3858	0.808	0.7659	0.7638	0.9758
Swin Transformer	0.3982	0.3948	0.8241	0.7479	0.7467	0.9780
ConvNeXt	0.2291	0.2225	0.7391	0.6925	0.6914	0.9690
FocalNet	0.5385	0.523	0.8715	0.7728	0.7714	0.9739
UniFormer	0.3655	0.3631	0.7964	0.6494	0.6476	0.9648
SGFormer	0.4329	0.3954	0.8337	0.7241	0.7181	0.9766
BiFormer	0.4911	0.4908	0.8451	0.7081	0.7067	0.9561
FasterNet	0.3115	0.3038	0.8176	0.7306	0.7295	0.9717
StarNet	0.4454	0.4437	0.8089	0.6823	0.6800	0.9512
HiFuse	0.3959	0.3876	0.8188	0.7714	0.7695	0.9806
StarMA Net	**0.6685**	**0.6640**	**0.9134**	**0.8610**	**0.8614**	**0.9857**

The bolded items are the best indicators.

##### Ablation study

We select StarMA Net as the benchmark model and demonstrate the effectiveness of the proposed method on the Kvasir dataset through ablation experiments that investigate replacement attention mechanisms. During the experiment, the experimental environment and parameter settings remain consistent with the above experiments. Table 7 provides a summary of the performance of our proposed method in comparison with other advanced models on the Kvasir dataset. The results indicate that the attention mechanism incorporated into our baseline model performs well on this dataset. Notably, our proposed StarMA mechanism outperforms other attention mechanisms in key performance metrics. This demonstrates that StarMA can effectively capture subtle differences and crucial information within the focal areas of the image, thereby enabling the model to concentrate more accurately on the target regions.

**Table 7:** Different attention mechanisms were employed for ablation studies on the Kvasir dataset

Model	Accuracy	Precision	Recall	F1-score
SA	0.8583	0.8617	0.8583	0.8577
CBAM	0.8533	0.8610	0.8533	0.8524
ECA	0.8550	0.8630	0.8550	0.8540
Ca	0.8683	0.8711	0.8683	0.8678
NAM	0.8600	0.8647	0.8600	0.8593
EMA	0.8650	0.8705	0.8650	0.8642
StarMA	**0.8783**	**0.8804**	**0.8783**	**0.8778**

The bolded items are the best indicators.

Meanwhile, we also conducted ablation experiments on each sub-module in StarMA Net to verify the effectiveness of each sub-module. The experimental results are shown in Table 8.

**Table 8:** Ablation research of each sub-module of StarMA Net

CCIB	SSS	SCB	CSFA	Accuracy	Precision	Recall	F1-score
✓	✓	–	–	0.8533	0.8544	0.8533	0.8533
–	–	✓	–	0.8583	0.8628	0.8583	0.8576
✓	✓	✓	–	0.8633	0.8663	0.8633	0.8626
✓	–	✓	✓	0.8650	0.8694	0.8650	0.8652
✓	✓	✓	✓	**0.8783**	**0.8804**	**0.8778**	**0.8778**

The bolded items are the best indicators.

As can be seen from the table, when the attention mechanism only uses the SCB or the CCIB module, the classification accuracy is 0.8583 or 0.8533, respectively, which is lower than the classification effect of the model. However, compared with other classification models, it still has a good classification effect. When the SCB and CCIB dual-branch attention mechanisms are employed, the model achieves a notable improvement over the single-branch attention mechanism, attaining a classification accuracy of 0.8650 and a classification precision of 0.8626. Compared with the model without CSFA, the accuracy of the attention mechanism without the SSS module reached 0.8650 and the F1 score was also higher.

#### Complexity analysis

Furthermore, we calculated the parameter count, FLOPs, and inference speed of StarMA Net and summarized all the comparison models. It can be seen from Table 9 that the parameter scale of StarMA Net is approximately 47.5M, which is much smaller than models such as ViT-Base or Swin-B. Meanwhile, it shows better accuracy and generalization in medical image tasks, proving its good practicability and cost performance. At the same time, it achieves a good balance between computational overhead and classification performance. Although it is not the lightest model, it can significantly improve accuracy while maintaining an acceptable complexity, which is of great significance for medical image analysis scenarios.

**Table 9:** Analysis of computational efficiency

Model	Image size	params	FLOPs	Average inference time
VGG	224^2^	143.67M	19.63G	7.09 ms
ResNet	224^2^	22M	3.7G	2.91 ms
EfficientNet	224^2^	12M	1.02G	10.21 ms
Vision Transformer	224^2^	86M	17.6G	8.76 ms
Swin Transformer	224^2^	88M	15.4G	18.50 ms
ConvNeXt	224^2^	89M	15.4G	8.93 ms
FocalNet	224^2^	88.1M	15.3G	9.88 ms
UniFormer	224^2^	49.78M	7.77G	18.89 ms
BiFormer	224^2^	57M	9.8G	26.13 ms
SGFormer	224^2^	77.64M	14.99G	49.70 ms
FasterNet	224^2^	15M	1.91G	3.13 ms
StarNet	224^2^	5.75M	0.76G	5.31 ms
HiFuse	224^2^	132.31M	20.24G	14.00 ms
StarMA Net	224^2^	47.5M	18.11G	8.53 ms

The original design intention of this study is to evaluate the feature representation ability and generalization performance of the proposed model itself without relying on additional regularization or data augmentation. Some datasets have a relatively small sample size, but the image complexity is high and the differences are large. A sufficient number of model parameters are required for effective fitting, so there is inevitably a certain risk of overfitting.

To further verify the rationality of the model complexity design, we conducted a channel reduction experiment, halving the number of model channels, and retrained it on the COVID-19 dataset. The results show that after the channel halving, the overall accuracy of the model decreased from 0.7241 to 0.7094, the F1-score decreased by approximately 3.3 percentage points, the recall rate decreased significantly, while the precision and specificity improved slightly.

This experiment indicates that the model complexity design of StarMA-Net is appropriate and can achieve good feature fitting and generalization performance on small-sample and complex medical image datasets. The performance declined after the channel was halved, indicating that an appropriate model capacity is necessary for capturing fine-grained lesion features. Therefore, this model strikes a good balance between computational complexity and recognition accuracy, and it has the ability to perform stably on small datasets. The comparison results are shown in Table 10.

**Table 10:** Complexity analysis experiment

Model	Accuracy	Precision	Recall	F1-score
The number of channels of StarMA Net is halved	0.7094	0.7556	0.6476	0.6974
StarMA Net	0.7241	0.7379	0.7238	0.7245

In addition, we conducted a quantitative analysis of the star-shaped structure in enhancing channel interaction to verify its effectiveness. Specifically, we calculated the variance of the channel coupling index (CCI) and the channel attention weights. The results show that after the introduction of the SSS module, the CCI significantly increased (0.0092 → 0.0198, a 115% increase), indicating a stronger dependence between channels. Meanwhile, the variance of channel weights has also increased, indicating that the responses among different channels are more differentiated. These findings provide strong quantitative evidence and further theoretical support for the role of star-shaped structures in enhancing channel interactions. The relevant experiments are shown in Table 11.

**Table 11:** Quantitative analysis of the SSS module at the channel interaction level

Model	Average CCI of the test set	The average pooled channel variance of the test set
No SSS	0.0092	0.012542 ± 0.007090
StarMA net	0.0198	0.023966 ± 0.024171

#### StarMA visualization

**Figure 9:**
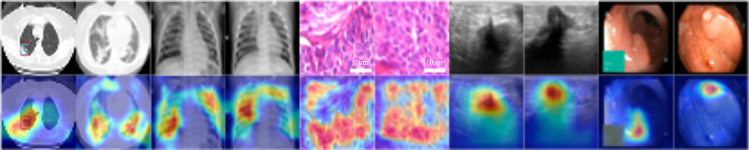
The Grad-CAM visual results of StarMA Net on medical image datasets of five different modalities The scale of the pathological image in the figure is used as an approximate reference.

To further illustrate the information capture capabilities of the proposed StarMA mechanism, we used the Grad-CAM^55^ method to visualize the model and react to the regions of interest in the model in the form of thermal maps. Figure 9 presents the visual results of this method applied to five different modality datasets.

It is evident that StarMA responds to higher calorific value in the lesion area and accurately covers the lesion area. This experiment demonstrates that our proposed StarMA mechanism effectively integrates features across different scales, captures subtle differences and critical information within focal regions, and enhances the model’s focus on regions of interest, thereby improving classification performance.

## Discussion

The StarMA module employs a parallel substructure strategy, using multi-branch parallel subnetworks to establish both short-term and long-term dependencies to adapt to the lesion areas in various images. Simultaneously, precise spatial structure information is retained within the channels to enhance image feature representation and capture broader contextual information. The star-shaped operation maps the input to a high-dimensional, nonlinear feature space without expanding the network, thereby improving the model’s representational capability. The integration of context information at different scales enables the network to achieve better pixel-level attention to high-level feature maps, allowing the model to more effectively capture subtle differences and critical information, thus enhancing classification performance for medical images.

The proposed StarMA Net is a visual backbone based on StarMA, exhibiting outstanding classification performance across five distinct medical imaging modalities: COVID-19-CT, Breast ×40, Chest X-ray, BUSI, and Kvasir. This verifies the validity and generalizability of our proposed model.

In this paper, we propose a StarMA mechanism to enhance image feature representation, generate more recognizable feature representations, and improve the classification performance of models on medical images. The algorithm employs multi-scale parallel subnetworks to establish both short-term and long-term dependencies, effectively utilizing spatial information to capture subtle differences and critical details in focal areas without reducing the channel dimension. Based on this attention mechanism, we designed a visual backbone and conducted experiments on datasets with various imaging methods to evaluate the feature representation and generalization ability of the model. Extensive experimental results demonstrate that our algorithm exhibits excellent robustness and generalization ability across various modal medical datasets, consistently maintaining high classification performance. We anticipate that this work could play an important role in a variety of downstream tasks involving medical images across different modalities.

### Limitations of the study

Of course, our experiment also has limitations. Our current research only focuses on the medical image field of the above five diagnostic datasets and has not yet expanded to study other medical images and natural images. We believe that exploring how the StarMA mechanism performs with large medical and natural image datasets could be a promising direction for future research. On the other hand, we plan to extend StarMA Net toward 3D feature modeling and cross-modal fusion to better align with clinical practice. Specifically, 3D convolutions or slice-level aggregation strategies can be employed to capture volumetric dependencies in magnetic resonance imaging/CT data, while cross-attention-based multi-branch architectures may facilitate integration of imaging and clinical text features. As these extensions inevitably increase computational cost, lightweight modules, patch-based training, and progressive optimization strategies will be considered to strike a balance between performance and efficiency. Furthermore, due to the limitations of current data acquisition, our current research is limited to medical image classification, and there are also relatively few related images, including those of low quality and those with overlapping lesions. We will collaborate closely with the hospital in the subsequent work, further exploring its value in clinical applications and investigating the application of the model in other downstream tasks, such as segmentation and object detection.

## Resource availability

### Lead contact

Requests for further information and resources should be directed to and will be fulfilled by the lead contact, Juan Wang (wj20221213@126.com).

### Materials availability

This study did not generate new unique reagents.

### Data and code availability

• All data reported in this paper will be shared by the lead contact upon request.
• The original code can be obtained from the lead contact as upon request.
• Any additional information required to reanalyze the data reported in this paper is available from the lead contact upon request.

## Acknowledgments

This work was supported partly by General Program of 10.13039/501100001809National Natural Science Foundation of China (nos. 62176217), supported by the Innovation Team Funds of 10.13039/501100007433China West Normal University (grant nos. KCXTD2022-3), in part supported by the Chinese Government Guidance Fund on Local Science and Technology Development of Sichuan Province under grant 2024ZYD0272, and supported by the Intelligent Medicine and Health Big Data Key Laboratory of Sichuan Province (grant no. ZNYX2505).

## Author contributions

J.C., conceptualization, methodology, software, investigation, validation, visualization, writing – original draft, and writing– review & editing; J.L., investigation and validation; X.L., investigation and validation; S.L., validation and data curation; J.W., funding acquisition, supervision, and project administration; B.Z., resources.

## Declaration of interests

The authors declare no competing interests.

## STAR★Methods

### Key resources table

**Table:** 

REAGENT or RESOURCE	SOURCE	IDENTIFIER
**Datasets**		

COVID-19 CT	He et al.^40^	https-github.com-UCSD-AI4H-COVID-CT/Images-processed at master · desaisrkr/https-github.com-UCSD-AI4H-COVID-CT · GitHub
BreakHis	Spanhol et al.^41^	BreakHis (kaggle.com)
Chest X-ray	Kermany et al.^42^	Chest X-Ray Images (Pneumonia) (kaggle.com)
BUSI	Dhabyani et al.^43^	Breast Ultrasound Images Dataset (kaggle.com)
Kvasir	Pogorelov et al.^44^	Kvasir Dataset (kaggle.com)

**Software and algorithms**		

Python (version 3.9.0)	Python software	https://www.python.org/
PyTorch (version 1.12.0)	Python library	https://pytorch.org/
PyCharm version (2023.1.2)	Python IDE	https://www.jetbrains.com/pycharm/

### Experimental model and study participant details

The datasets used in this experiment are all public datasets, and it does not involve clinical experiments.

#### Data sets

##### COVID-19 CT

The COVID-19 CT dataset comprises 746 CT scan images, including 349 positive cases for COVID-19 and 397 negative or indicative of other diseases. Following the partitioning method described in the literature, the dataset is divided into training, validation, and test sets.

#### BreakHis 400X

The Breast Cancer Histopathological Image Classification (BreakHis) dataset comprises 9,109 microscopic images of breast tumor tissue from 82 patients. It is categorized into benign and malignant tumors and includes images at four magnifications: 40X, 100X, 200X, and 400X, totaling 2,480 benign and 5,429 malignant samples. For this experiment, we used the 400X magnification subset, which includes 588 benign and 1,232 malignant samples, totaling 1,820 images. The dataset was divided into training, validation, and test sets in a 7:1.5:1.5 ratio.

#### Chest X-ray

The pneumonia Chest X-ray dataset consists of two types of images: pneumonia and normal. The dataset contains a total of 5,856 images, including 1,583 normal and 4,273 pneumonia cases (2,780 bacterial and 1,493 viral). The dataset is partitioned into training, validation, and test sets in a 7:1.5:1.5 ratio.

##### BUSI

The Breast Ultrasound Images Dataset (BUSI) comprises ultrasound images of breast tissue from women aged 25 to 75, collected in 2018. It includes 600 patients and a total of 780 images, each with an average size of 500 × 500 pixels. The images are categorized into three classes: 133 normal, 437 benign, and 210 malignant. For experimentation, the dataset was split into training, validation, and test sets in a 7:1.5:1.5 ratio.

##### Kvasir

The dataset comprises 4,000 endoscopic images of gastrointestinal disorders, categorized into eight groups, each containing 500 images. It encompasses a range of images featuring anatomical markers (e.g., Z-line, pylorus, cecum) and pathological findings (e.g., esophagitis, polyps, ulcerative colitis). The dataset is partitioned into training, validation, and test sets with a 7:1.5:1.5 ratio.

The number of datasets and their division methods are shown in Datasets Table.

**Table:** Datasets table

Experimental environment configuration	The number of datasets	Division ratio
COVID-19 CT	746	7:1.5:1.5
BreakHis 400X	1,820	7:1.5:1.5
Chest X-ray	5,856	7:1.5:1.5
BUSI	780	7:1.5:1.5
Kvasir	4,000	7:1.5:1.5

### Method details

#### Training settings

The experimental platform is configured as follows: an NVIDIA GeForce RTX 3060 12GB GPU and 16GB (8GB x 2) of memory. The code is written in Python 3.9, utilizing PyTorch^56^ as the deep learning library and CUDA version 12.0. During training, the learning rate is set to 1e-5, the batch size is 16, the AdamW optimizer is used, and the cross-entropy loss function is employed. To ensure fairness in comparisons, the Drop Path value was set to 0. To minimize random errors, no online data augmentation methods, such as random rotation or cropping, were applied. Input images were resized to 224 × 224 pixels using bilinear interpolation. Each model was trained for 100 iterations, and the weights of the model with the highest validation accuracy were saved for testing. Detailed parameter Settings are shown in Experimental Setting Table.

**Table:** Experimental platform table.

Experimental environment configuration	Version number
Graphics card	NVIDIA GeForce RTX 3060 12GB GPU
Memory stick	16GB (8GB x 2) of memory.
CUDA	12.0

**Table:** Experimental setting table.

Training config	Parameter value
Image size	224 × 224
Optimizer	Adam
Learning Rate	1e-5
Batch size	16
Epoch	100
Online data enhancement	No
Drop path rate	0

### Quantification and statistical analyses

#### Evaluation metrics formulas

We used accuracy, precision, sensitivity, specificity, F1 score, MCC, Kappa, and AUC as evaluation metrics to evaluate the performance of our proposed methods. Accuracy measures the proportion of correctly classified samples out of the total number of samples and is the most used metric for assessing classification performance. Precision, also known as positive predictive value, represents the proportion of correctly predicted positive samples among all predicted positives. Sensitivity, which is equivalent to Recall, indicates the proportion of true positive samples correctly identified out of the total true positives. Specificity measures the proportion of correctly classified negative cases out of the total number of negatives. The F1 score is the harmonic mean of precision and recall, providing a comprehensive view of the model's predictive power. A higher F1 score indicates a more effective test method. Accuracy, precision, sensitivity, specificity, and F1 scores are defined by Eq:

$$\mathrm{Accuracy}=\frac{\mathrm{TP}+\mathrm{FN}}{\mathrm{TP}+\mathrm{TN}+\mathrm{FP}+\mathrm{FN}}$$

$$\mathrm{Pecision}=\frac{\mathrm{TP}}{\mathrm{TP}+\mathrm{FP}}$$

$$\mathrm{Sensitivity}=\frac{\mathrm{TP}}{\mathrm{TP}+\mathrm{FN}}$$

$$\mathrm{Specificity}=\frac{\mathrm{TN}}{\mathrm{FP}+\mathrm{TN}}$$

$$F1\;\mathrm{Score}=\frac{2\times \mathrm{precision}\times \mathrm{recall}}{\mathrm{precision}+\mathrm{recall}}$$

The Matthews correlation coefficient (MCC) is primarily used to assess binary classification problems. Its value ranges from -1 to 1, where 1 indicates a perfect prediction, 0 signifies a random prediction, and -1 denotes a completely incorrect prediction. The MCC is calculated as follows:

$$MCC=\frac{TP\times TN-FP\times FN}{\sqrt{\left(TP+FP\right)\left(TP+FN\right)\left(TN+FP\right)\left(TN+FN\right)}}$$

TP (True Positive): The number of positive samples (foreground pixels) correctly classified as positive.

FP (False Positive): The number of negative samples (background pixels) incorrectly classified as positive.

TN (True Negative): The number of negative samples (background pixels) correctly classified as negative.

FN (False Negative): The number of positive samples (foreground pixels) incorrectly classified as negative.

The Kappa coefficient is another metric used to evaluate the performance of classification models by measuring the agreement between predicted and observed classifications. It ranges from 0 to 1, where 1 indicates complete agreement and 0 indicates agreement by chance. The Kappa coefficient is calculated using the following formula:

$$Kappa=\frac{\left(P_{0}-P_{e}\right)}{1-P_{e}}$$

*P*_0_ represents the observed consistency, which is the actual accuracy. *P*_*e*_ represents the random consistency, which is the accuracy achieved by random guessing.

The Area Under the Receiver Operating Characteristic Curve (AUC-ROC) is also a crucial metric for evaluating models. It is plotted with the False Positive Rate (FPR) on the x-axis and the Taxis.^57^ A curve closer to the point (0, 1) indicates a better discriminative ability of the model.

## References

[1] Ghosh S., Das S. (2024). Multi-scale morphology-aided deep medical image segmentation. Eng. Appl. Artif. Intell..

[2] Kumar A., Kim J., Lyndon D., Fulham M., Feng D. (2017). An ensemble of fine-tuned convolutional neural networks for medical image classification. IEEE J. Biomed. Health Inform..

[3] Jiang H., Diao Z., Shi T., Zhou Y., Wang F., Hu W., Zhu X., Luo S., Tong G., Yao Y.D. (2023). A review of deep learning based multiple-lesion recognition from medical images: classification, detection and segmentation. Comput. Biol. Med..

[4] Vaswani A., Shazeer N., Parmar N., Uszkoreit J., Jones L., Gomez A.N., Kaiser Ł., Polosukhin I. (2017). Attention is all you need. Adv. Neural Inf. Process. Syst..

[5] He X., Tan E.-L., Bi H., Zhang X., Zhao S., Lei B. (2022). Fully transformer network for skin lesion analysis. Med. Image Anal..

[6] Ouyang D., He S., Zhang G., Luo M., Guo H., Zhan J., Huang Z. (2023). 2023 IEEE International Conference on Acoustics, Speech and Signal Processing, ICASSP.

[7] Liu S., Wang L., Yue W. (2024). An efficient medical image classification network based on multi-branch CNN, token grouping Transformer and mixer MLP. Appl. Soft Comput..

[8] Ma X., Dai X., Bai Y., Wang Y., Fu Y. (2024). 2024 IEEE/CVF Conference on Computer Vision and Pattern Recognition.

[9] Liu Z., Mao H., Wu C., Feichtenhofer C., Darrell T., Xie S. (2022). 2022 IEEE/CVF Conference on Computer Vision and Pattern Recognition.

[10] Dosovitskiy A., Beyer L., Kolesnikov A., Weissenborn D., Zhai X., Unterthiner T., Dehghani M., Minderer M., Heigold G., Gelly S. (2020). An Image is Worth 16x16 Words: Transformers for Image Recognition at Scale. arXiv.

[11] Chollet F. (2017). 2017 IEEE/CVF Conference on Computer Vision and Pattern Recognition.

[12] Chen P.W., Tseng B.Y., Yang Z.H., Yu C.H., Lin K.T., Chen J.N., Liu P.Y. (2024). Deep learning model for diagnosis of venous thrombosis from lower extremity peripheral ultrasound imaging. iScience.

[13] Wang Z., Luo S., Chen J., Jiao Y., Cui C., Shi S., Yang Y., Zhao J., Jiang Y., Zhang Y. (2024). Multi-modality deep learning model reaches high prediction accuracy in the diagnosis of ovarian cancer. iScience.

[14] Cheng J., Tian S., Yu L., Gao C., Kang X., Ma X., Wu W., Liu S., Lu H. (2022). ResGANet: Residual group attention network for medical image classification and segmentation. Med. Image Anal..

[15] Cao R., Liu Y., Wen X., Liao C., Wang X., Gao Y., Tan T. (2024). Reinvestigating the performance of artificial intelligence classification algorithms on COVID-19 X-Ray and CT images. iScience.

[16] Jin L., Sun T., Liu X., Cao Z., Liu Y., Chen H., Ma Y., Zhang J., Zou Y., Liu Y. (2023). A multi-center performance assessment for automated histopathological classification and grading of glioma using whole slide images. iScience.

[17] Wei H.L., Wei C., Feng Y., Yan W., Yu Y.-S., Chen Y.-C., Yin X., Li J., Zhang H. (2023). Predicting the efficacy of non-steroidal anti-inflammatory drugs in migraine using deep learning and three-dimensional T1-weighted images. iScience.

[18] Kora P., Ooi C.P., Faust O., Raghavendra U., Gudigar A., Chan W.Y., Meenakshi K., Swaraja K., Plawiak P., Rajendra Acharya U. (2022). Transfer learning techniques for medical image analysis: A review. Biocybern. Biomed. Eng..

[19] Shen W., Zhou M., Yang F., Yu D., Dong D., Yang C., Zang Y., Tian J. (2017). Multicrop convolutional neural networks for lung nodule malignancy suspiciousness classification. Pattern Recogn..

[20] Sun J., Wu B., Zhao T., Gao L., Xie K., Lin T., Sui J., Li X., Wu X., Ni X. (2023). Classification for thyroid nodule using ViT with contrastive learning in ultrasound images. Comput. Biol. Med..

[21] Gheflati B., Rivaz H. (2022). 2022 44th Annual International Conference of the IEEE Engineering in Medicine & Biology Society.

[22] Sha Y., Zhang Q., Zhai X., Hou M., Lu J., Meng W., Wang Y., Li K., Ma J. (2024). CerviFusionNet: A multi-modal, hybrid CNN-transformer-GRU model for enhanced cervical lesion multi-classification. iScience.

[23] Li P., Hu Y. (2024). Deep magnetic resonance fingerprinting based on Local and Global Vision Transformer. Med. Image Anal..

[24] Ding Y., Yi Z., Xiao J., Hu M., Guo Y., Liao Z., Wang Y. (2024). CTH-Net: A CNN and Transformer hybrid network for skin lesion segmentation. iScience.

[25] Li Z., Cong Y., Chen X., Qi J., Sun J., Yan T., Yang H., Liu J., Lu E., Wang L. (2023). Vision transformer-based weakly supervised histopathological image analysis of primary brain tumors. iScience.

[26] Zhou J., Wang X., Niu R., Shang X., Wen J. (2024). Predicting circRNA-miRNA interactions utilizing transformer-based RNA sequential learning and high-order proximity preserved embedding. iScience.

[27] Xu Z., Guo X., Wang J. (2024). Enhancing skin lesion segmentation with a fusion of convolutional neural networks and transformer models. Heliyon.

[28] Woo S., Park J., Lee J., Kweon I. (2018). 2018 European Conference on Computer Vision.

[29] Wang Q., Wu B., Zhu P., Li P., Zuo W., Hu Q. (2020). 2020 IEEE/CVF Conference on Computer Vision and Pattern Recognition.

[30] Zhang Q., Yang Y. (2021). 2021 IEEE International Conference on Acoustics, Speech and Signal Processing, ICASSP.

[31] Liu Y., Shao Z., Teng Y., Hoffmann N. (2021). NAM: Normalization-based Attention Module. arXiv.

[32] Hou Q., Zhou D., Feng J. (2021). 2021 IEEE/CVF Conference on Computer Vision and Pattern Recognition.

[33] Hu J., Shen L., Sun G. (2018). 2018 IEEE/CVF Conference on Computer Vision and Pattern Recognition.

[34] Guo M.H., Lu C.Z., Liu Z.N., Cheng M.M., Hu S.M. (2023). Visual attention network. Comput. Vis. Media (Beijing)..

[35] Liu W., Sun J., Li H., Wang Y., Wang Z. (2025). CSEA-Net: A channel–spatial enhanced attention network for lung tumor segmentation on CT images. iScience.

[36] Xiang H., Shen J., Yan Q., Xu M., Shi X., Zhu X. (2023). Multi-scale representation attention based deep multiple instance learning for gigapixel whole slide image analysis. Med. Image Anal..

[37] Tang L., Tian C., Yang H., Cui Z., Hui Y., Xu K., Shen D. (2023). TS-DSANN: Texture and shape focused dual-stream attention neural network for benign-malignant diagnosis of thyroid nodules in ultrasound images. Med. Image Anal..

[38] Fan Z., Gong P., Tang S., Lee C.U., Zhang X., Song P., Chen S., Li H. (2023). Joint localization and classification of breast masses on ultrasound images using an auxiliary attention-based framework. Med. Image Anal..

[39] Raja S., Luo T. (2022). 2022 Machine Learning and Knowledge Discovery in Databases.

[40] He X., Yang X., Zhang S., Zhao J., Zhang Y., Xing E., Xie P. (2020). Sample-Efficient Deep Learning for COVID-19 Diagnosis Based on CT Scans. medRxiv.

[41] Spanhol F.A., Oliveira L.S., Petitjean C., Heutte L. (2016). A Dataset for Breast Cancer Histopathological Image Classification. Ieee T Bio-Med Eng..

[42] Kermany D.S., Goldbaum M., Cai W., Valentim C.C.S., Liang H., Baxter S.L., McKeown A., Yang G., Wu X., Yan F. (2018). Identifying medical diagnoses and treatable diseases by image-based deep learning. Cell.

[43] Al-Dhabyani W., Gomaa M., Khaled H., Fahmy A. (2020). Dataset of breast ultrasound images. Data Brief.

[44] Pogorelov K., Randel K., Griwodz C., Eskeland S., Lange T., Johansen D., Spampinato C., Dang-Nguyen D., Lux M., Schmidt P. (2017). 2017 Proceedings of the 8th ACM on Multimedia Systems Conference.

[45] Simonyan K., Zisserman A. (2014). Very deep convolutional networks for large-scale image recognition. arXiv.

[46] Liu Z., Lin Y., Cao Y., Hu H., Wei Y., Zhang Z., Lin S., Guo B. (2021). Swin Transformer: Hierarchical Vision Transformer Using Shifted Windows, 2021 IEEE/CVF International Conference on Computer Vision. ICCV.

[47] Tan M., Le Q. (2019). EfficientNet: Rethinking model scaling for convolutional neural networks. arXiv.

[48] He K., Zhang X., Ren S., Sun J. (2016). 2016 IEEE Conference on Computer Vision and Pattern Recognition.

[49] Zhu L., Wang X., Ke Z., Zhang W., Lau R. (2023). 2023 IEEE/CVF Conference on Computer Vision and Pattern Recognition.

[50] Yang J., Li C., Dai X., Yuan L., Gao J. (2022). Focal Modulation Networks. arXiv.

[51] Li K., Wang Y., Zhang J., Gao P., Song G., Liu Y., Li H., Qiao Y. (2023). Uniformer: Unifying convolution and self-attention for visual recognition. IEEE Trans. Pattern Anal. Mach. Intell..

[52] Q. Wu, W. Zhao, C. Yang, H. Zhang, F. Nie, H. Jiang, Y. Bian, J. Yan, SGFormer: Simplifying and Empowering Transformers for Large-Graph Representations, 2023, Preprint at arXiv, 10.48550/arXiv.2306.10759.

[53] Chen J., Kao S., He H., Zhuo W., Wen S., Lee C., Chan S. (2016). 2023 IEEE/CVF Conference on Computer Vision and Pattern Recognition.

[54] Huo X., Sun G., Tian S., Wang Y., Yu L., Long J., Zhang W., Li A. (2024). HiFuse: Hierarchical multi-scale feature fusion network for medical image classification. Biomed. Signal Process Control.

[55] Selvaraju R., Cogswell M., Das A., Vedantam R., Parikh D., Batra D. (2017). 2017 IEEE/CVF International Conference on Computer Vision, ICCV.

[56] A. Paszke, S. Gross, F. Massa, A. Lerer, J. Bradbury, G. Chanan, T. Killeen, Z. Lin, N. Gimelshein, L. Antiga, et al., PyTorch: An imperative style, high-performance deep learning library, 2019, Preprint at arXiv, 10.48550/arXiv.1912.01703

[57] Liu K., Wang W., Wang R., Cui X., Zhang L., Yuan X., Li X. (2023). CDF-LS: Contrastive Network for Emphasizing Feature Differences with Fusing Long- and Short-Term Interest Features. Appl. Sci..

