# Supplementary material for: StarMA Net: A star-shape multi-scale attention network for medical imaging classification
Source: iScience. 2025 Nov 25;28(12):114214. doi: 10.1016/j.isci.2025.114214 (PMC12756611; doi:10.1016/j.isci.2025.114214)
Supplement: Document S1. Figures S1–S4 [file mmc1.pdf]

iScience, Volume *28*

Supplemental information

StarMA Net: A star-shape multi-scale attention network for medical imaging classification

Junyang Cao, Junrui Lv, Xuegang Luo, Siyu Lai, Juan Wang, and Bochuan Zheng

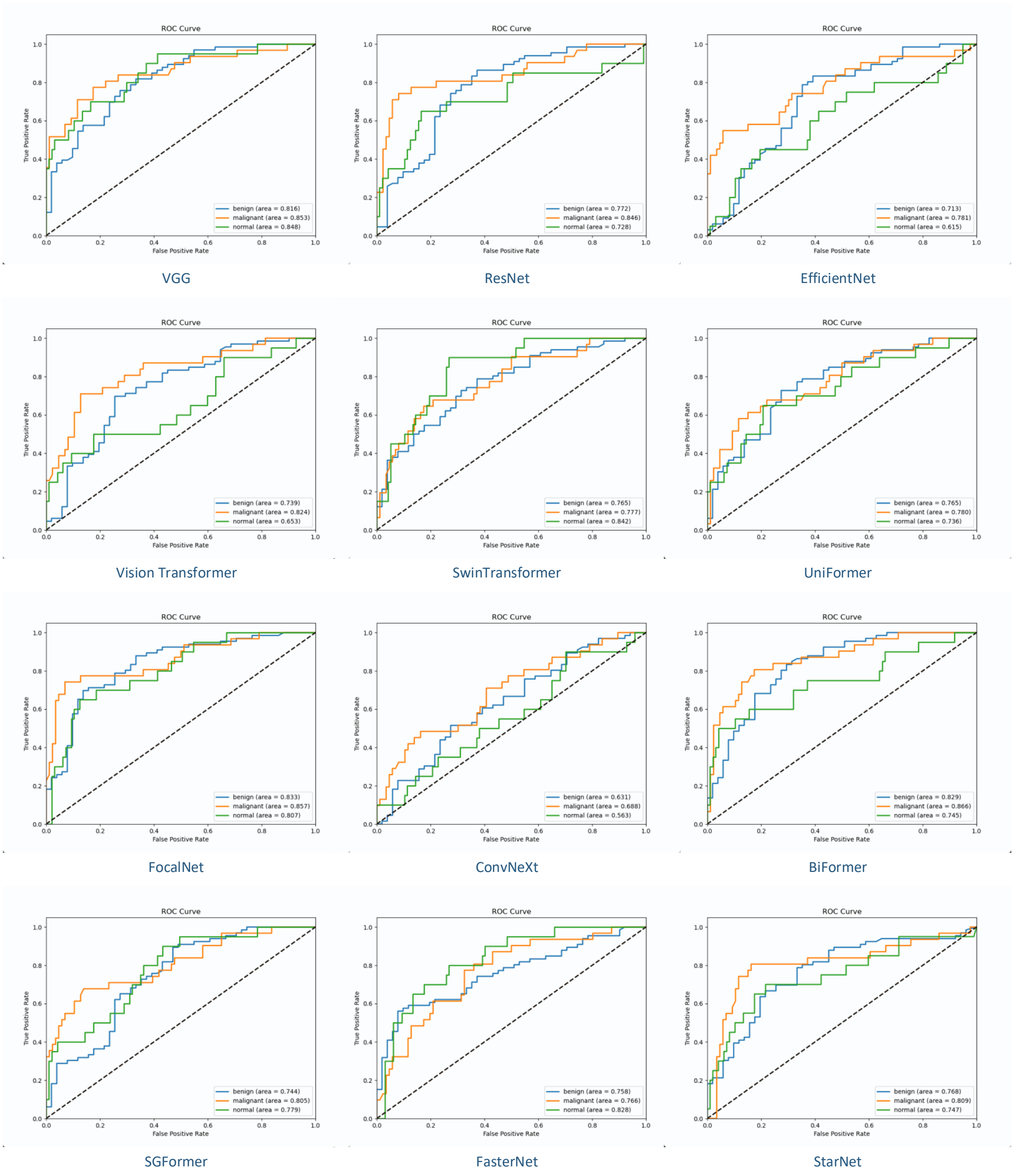

Fig.1. AUC-ROC curve on BUSI dataset

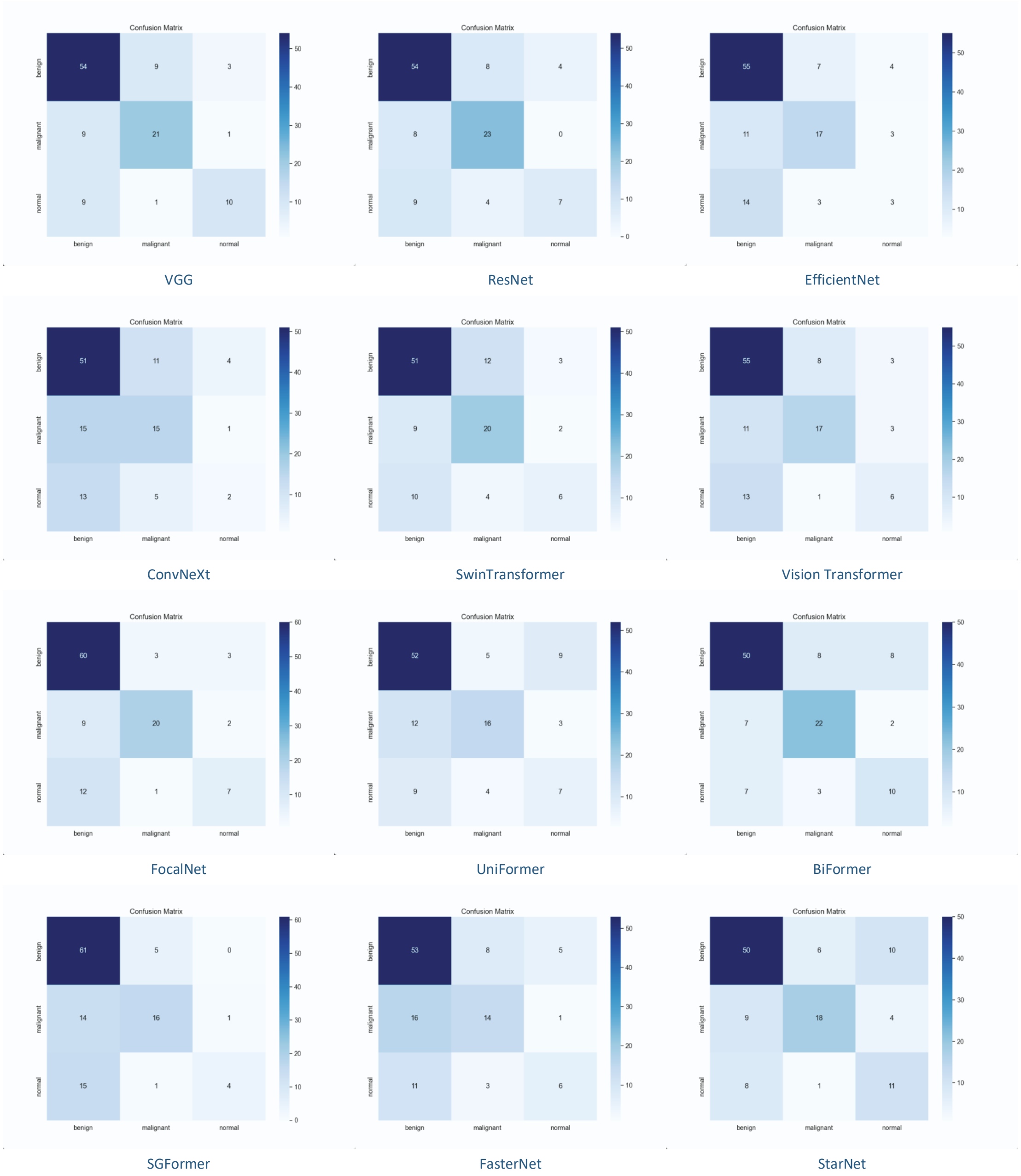

Fig.2. Confusion matrix on BUSI dataset.

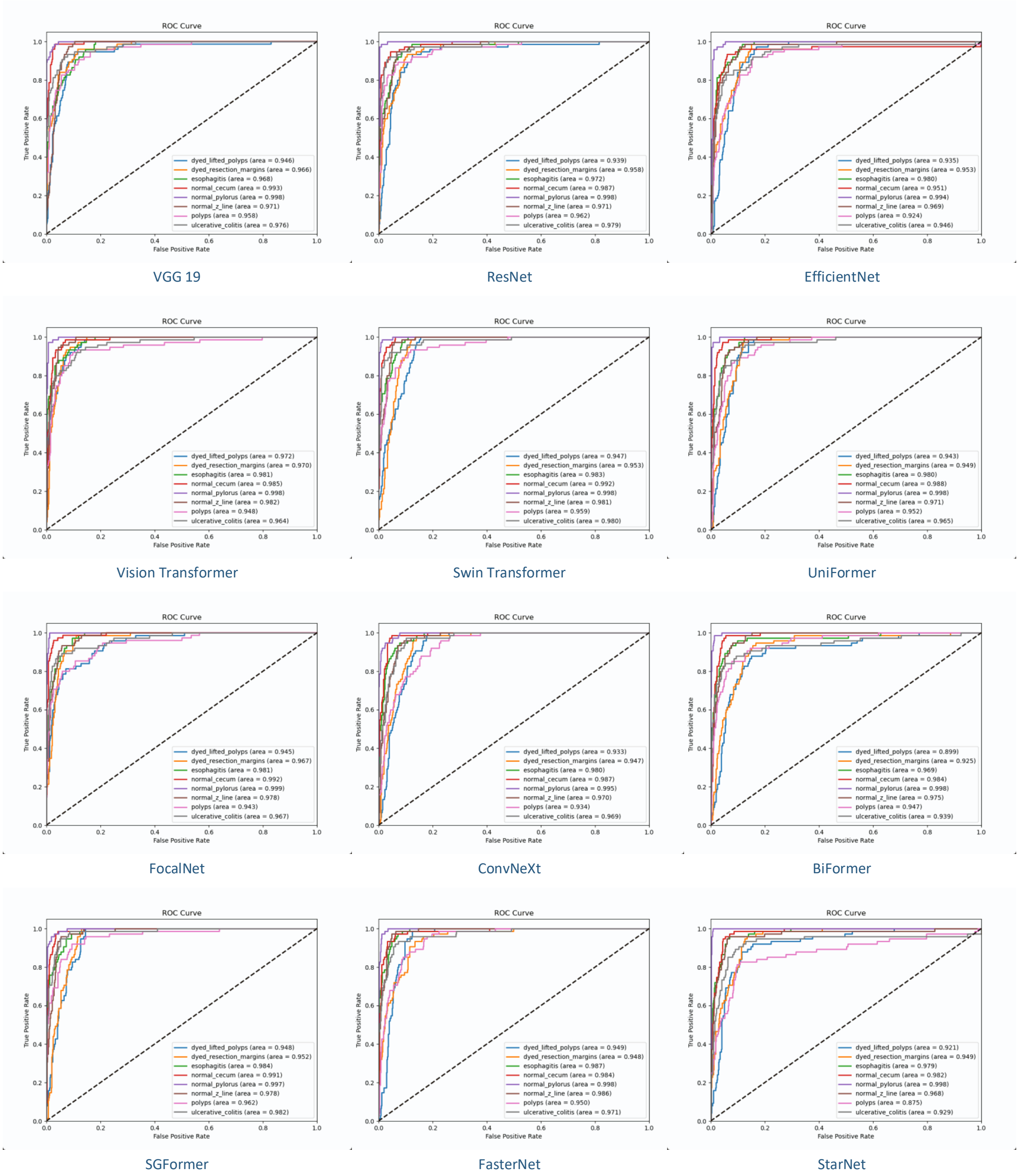

Fig.3. AUC-ROC curve on Kvasir dataset.

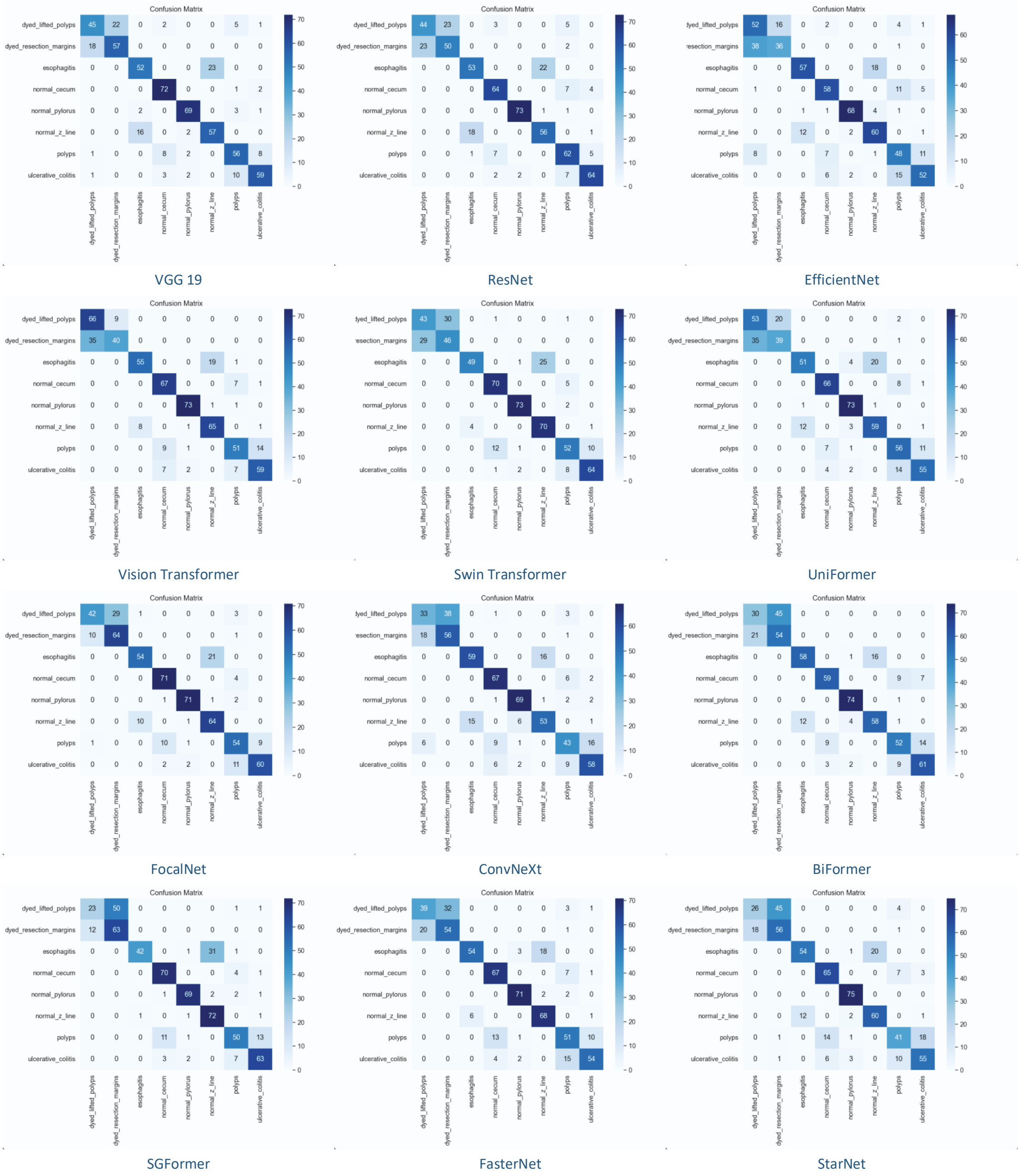

Fig.4. Confusion matrix on Kvasir dataset.
